# The Odonata of Quebec: Specimen data from seven collections

**DOI:** 10.3897/BDJ.8.e49450

**Published:** 2020-02-28

**Authors:** Colin Favret, Joseph Moisan-De Serres, Maxim Larrivée, Jean-Philippe Lessard

**Affiliations:** 1 University of Montreal, Montreal, Canada University of Montreal Montreal Canada; 2 Quebec Ministry of Agriculture, Fisheries and Food, Quebec City, Canada Quebec Ministry of Agriculture, Fisheries and Food Quebec City Canada; 3 Insectarium of Montreal, Montreal, Canada Insectarium of Montreal Montreal Canada; 4 Concordia University, Montreal, Canada Concordia University Montreal Canada

**Keywords:** Anisoptera, Canada, damselfly, distribution, dragonfly, natural history collection, specimen digitisation, Zygoptera

## Abstract

**Background:**

The Odonata, dragonflies and damselflies, constitute one of the more charismatic and better-studied orders of insects. The approximately 6,000 extant species on Earth can be variously found on all continents, except Antarctica. A relatively stable taxonomy, a relative ease of species identification and an aquatic immature stage has made the Odonata a taxon of interest in documenting the symptoms of global environmental change, especially at higher latitudes. The Odonata fauna of the north-temperate Canadian province of Quebec includes 150 species, many of which are at the northern limits of their geographic distribution.

**New information:**

Quebec hosts multiple entomological specimen depositories, including seven publicly-accessible research collections. One of these, the University of Montreal's Ouellet-Robert Entomological Collection, houses an exceptionally large collection of Odonata. An initial specimen data capture project for this collection gathered 31,595 Quebec Odonata occurrence records, but several Quebec species were missing and geographic coverage was biased towards the Montreal region. To complement this dataset, we undertook to digitise the Odonata records of six other public research collections. They are, in order of Quebec Odonata collection size, the Laval University Entomological Collection, McGill University's Lyman Entomological Museum, the Insectarium of Montreal Research Collection, the Quebec Government's Insect Collection, Bishop's University's Insect Collection and the Laurentian Forestry Centre's René-Martineau Insectarium. Of the 40,447 total specimen occurrence records, 36,951 are identified to the species level, including 137 of the 150 species officially-recorded in Quebec and 2 non-nominotypical subspecies. We here summarise the data and highlight the strengths and weaknesses of the datasets. The complete dataset is available with this publication (Suppl. material 1), whereas the specimen data associated with each collection are available as Darwin Core archives at Canadensys.net and will be updated as appropriate.

## Introduction

Dragonflies and damselflies (Insecta: Odonata) are large charismatic flying insects at the adult stage, aquatic naiads during their immature stages. Both adults and naiads are predatory, the former capturing their prey in flight, the latter using a distinctive extendable labial mask. Albeit generally less sensitive to water quality than mayflies, stoneflies and caddisflies (Ephemeroptera, Plecoptera and Trichoptera, respectively), Odonata naiads can be used as water quality indicators in some situations (Briers and Biggs 2003, Foote and Rice Hornung 2005, Osborn 2005). There are approximately 6,000 described species of Odonata (Dijkstra et al. 2013) and the conservation status of a large number of these is of concern (Gerlach et al. 2014, Clausnitzer and Jödicke 2004).

There are 150 species of Odonata recorded from the Canadian province of Quebec (151 listed by Savard (2019) minus the newly synonymised *Sympetrum janeae* Carle, 1993 (Paulson and Dunkle 2018, Pilgrim and Von Dohlen 2007)), representing 70% of the Canadian fauna (Cannings 2019) and one third of the species known from North America (Kalkman et al. 2007). Thanks to years of collection and study (Provancher 1871, Robert 1963, Pilon and Lagacé 1998, Hutchinson and Ménard 2014), the status of Quebec Odonata is relatively well-known (Cannings 2019). In places, there appears to be a replacement of specialist species by generalists (Piché and Hutchinson 2016), probably due to anthropogenic habitat change. In order to contribute to the Atlas of Quebec Odonata (Savard 2011), to promote the general knowledge of and future research on this group (e.g. Beatty et al. 2010, Grewe et al. 2012, Kalkman et al. 2018) and to facilitate the use of natural history museum data (e.g. Ball-Damerow et al. 2019, Lister 2011, Kharouba et al. 2018), we thought it important to digitise and make publicly accessible the specimen data in Quebec's public entomological collections.

### The Odonata collections of Quebec

**Figure 1.**
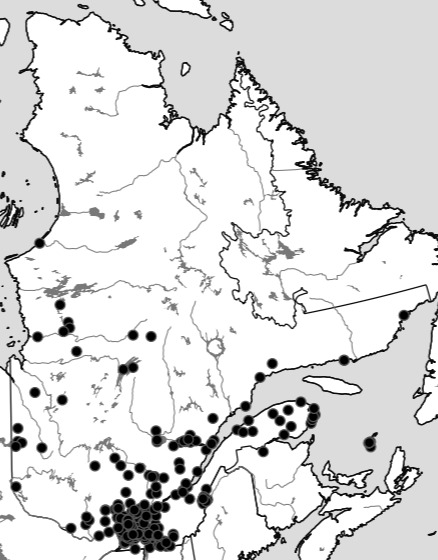
Collection localities of the Ouellet-Robert Collection specimens.

The Ouellet-Robert Entomological Collection at the University of Montreal has an exceptionally large holding of Odonata, so when the opportunity presented itself to digitise insect specimen data, this group was an obvious choice (Favret et al. 2019). Of the 33,122 Odonata specimen occurrence records in the Ouellet-Robert Collection, 31,595 (95%) are from the Canadian province of Quebec. However, of the 150 species of Odonata known from Quebec, the Ouellet-Robert Collection houses only 128 and their distribution records exhibit a "collection bias" (Ferro and Flick 2015) for the Montreal region (Fig. 1). Additionally, despite the large number of records, they are concentrated in a relatively small number of collection localities (222). In order to perform more rigorous distribution modelling and other computational analyses in the future, we sought to broaden the sampling, both geographically and taxonomically, by adding the specimen occurrence records from the other public research collections in Quebec. These include three other university collections and three governmental collections at the city, provincial and federal levels: McGill University's Lyman Museum, Bishop's and Laval Universities, the Insectarium of Montreal, the Government of Quebec and Natural Resources Canada's Laurentian Forestry Centre's Insectarium René Martineau (Table 1).

## Sampling methods

### Study extent

We targeted the Odonata held in seven public insect research collections of Quebec, but it should be noted that there are a number of other public teaching collections in the many universities and CÉGEPs (post-secondary, pre-university schools) in Quebec. In addition, the amateur entomologist community is organised and active in Quebec (Association des Entomologistes Amateurs du Québec 2020, Entomofaune du Québec 2020) and, given the popularity of Odonata collecting, a large amount of material, not catalogued here, is held in private collections.

### Sampling description

Data capture followed two distinct protocols. Prior to 2012 (Method 1), the Ouellet-Robert Collection specimen label data were parsed and captured manually and verbatim into an Excel spreadsheet. No photographs were taken. After import into a custom FileMaker Pro (FileMaker, Inc., Santa Clara, California, USA) relational database, locality georeferencing was conducted with reference to a downloaded gazeteer, the Canadian Geographical Names Data Base at Natural Resources Canada. Both the verbatim locality description and the gazeteer's locality name were recorded in the FileMaker database.

**Figure 2.**
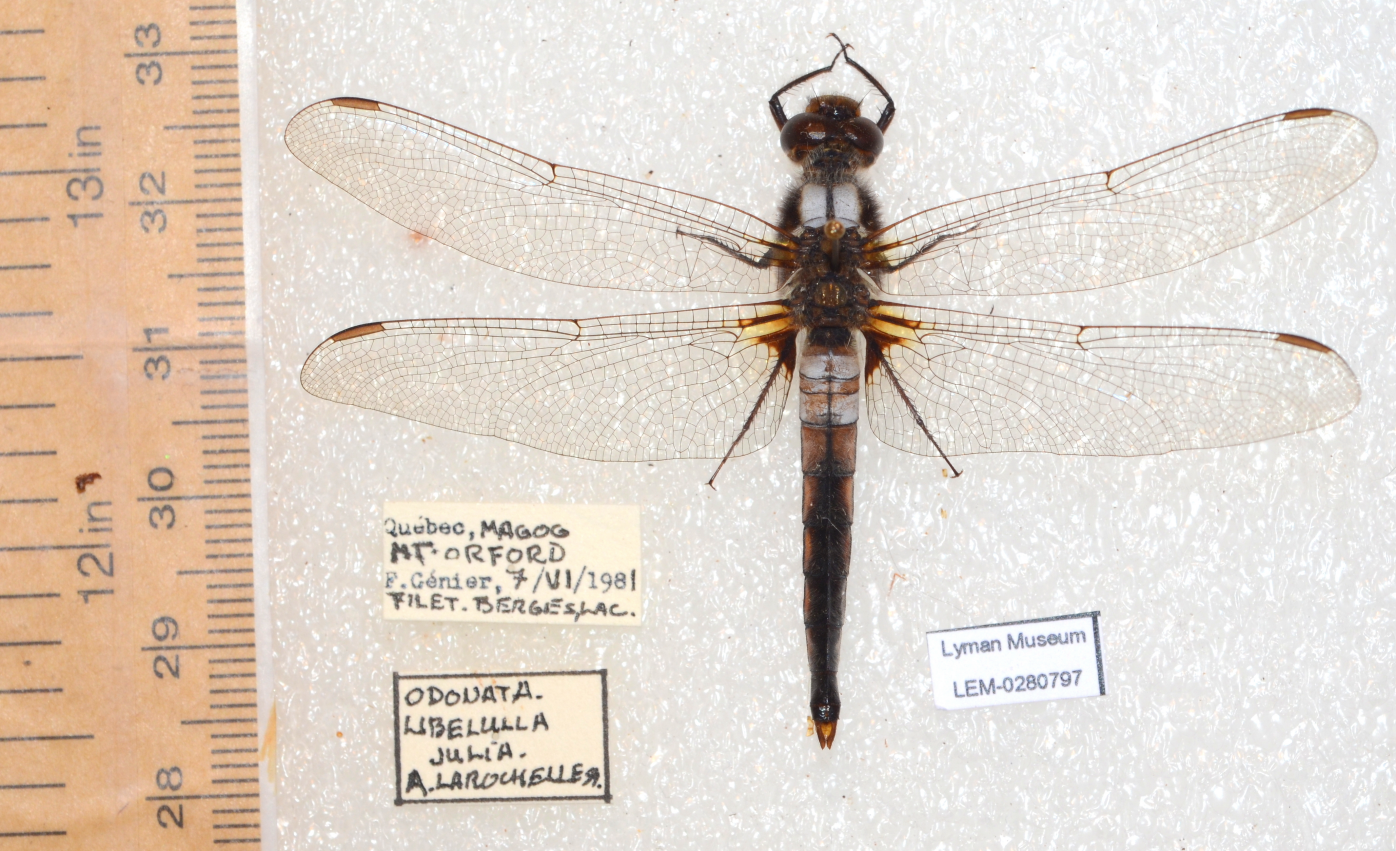
Example of pinned specimen photograph with labels removed.

**Figure 3.**
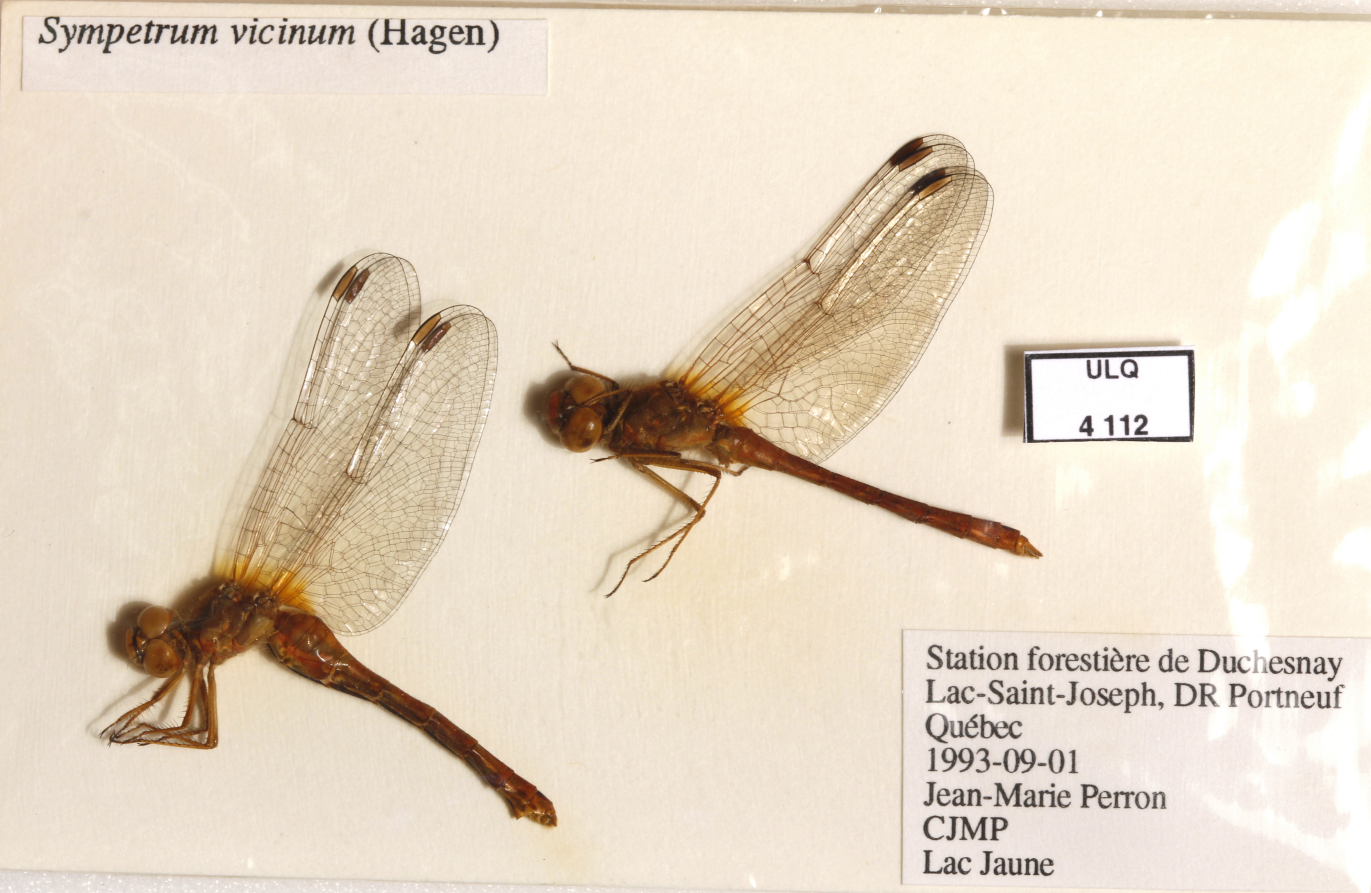
Example of specimens in glassine envelope, photographed in situ.

For the other six collections, after 2012 (Method 2), the digitisation process largely followed that described by Nelson et al. (2012). Labels of pinned specimens were removed and placed alongside the specimen, a unique identifier label was added and the ensemble was photographed (Fig. 2). Glassine envelopes were photographed as found, with the occasional displacement of the specimens in cases where they obstructed the labels (Fig. 3). Unfortunately, we were inconsistent in our use of callibration scale. We found that the rate of photography was optimal with three workers, the first preparing the specimens, the second photographing them, the third replacing them. With this set-up, we photographed an average of 1.2 pinned specimens and 4.0 envelopes per minute. Photograph files were renamed, either manually or with a simple perl script that added the collection code and sequential numbers, to correspond to each specimen's unique identifier, allowing for batch importation into a rapid-input FileMaker database.

In order to accelerate data input, only three numbers and one date were captured manually: 1) The taxon, based on the most recent determination, was captured with a reference ID to a nomenclator built on several taxonomic checklists (Paulson and Dunkle 2018, Garrison and von Ellenrieder 2016). 2) The collection locality, with its geoposition coordinates, was recorded with another reference ID to the same gazeteer mentioned above. Finally, 3) the collection date and 4) the number of specimens represented by each museum object (e.g. several specimens in a single glassine envelope (Fig. 3)) were recorded. Taxonomic data were added during batch import of the photographs. The other three data fields were added by hourly undergraduate employees, referencing the photos and the gazeteer. Their average data capture rate of 106 museum objects per hour was speeded up when multiple specimens in a row had been collected at the same locality.

Figures for the efficiency of the Ouellet-Robert Collection data capture are unavailable (Method 1). However, based on previous experience (Favret and Dewalt 2002), we estimate that it proceeded at approximately 12 museum objects per hour. On the other hand, photographing the museum objects first and then choosing to capture only the most critical data assured a higher rate (Method 2). Including photography, file naming and import and data capture, but excluding time for set-up, we averaged 19 objects per person-hour for pinned specimens, 44 for glassine envelopes. Although some data were not captured in the database, for example, the collector and determiner, these are available on the photographs and can be incorporated into the database in the future, as resources allow, without having to re-access the actual physical specimen.

### Quality control

The taxonomic nomenclature was referenced with the latest sources and is up-to-date. Additionally, most identifications were made by experts in Odonata taxonomy, most notably Adrien Robert and Jean-Marie Perron at the University of Montreal and Laval University, respectively. However, we did not re-identify every specimen and some of the taxonomy has changed since the original identifications, especially those of Robert. Notably, 663 specimens were identified as *Enallagma cyathigerum* (Charpentier, 1840), a species now known to be absent from North America. What was labelled as *E. cyathigerum* may properly be attributed the name *Enallagma annexum* (Hagen, 1861) (Turgeon et al. 2005). Alternatively, some specimens may actually be *Enallagma vernale* Gloyd, 1943, as this latter species was once considered a subspecies of *E. cyathigerum* (*Donnelly 1989*). Taxonomic determination is always subject to error and revision and users of the data should bear this in mind.

Likewise, the geographic latitude and longitude coordinates reference precise localities in Quebec. In most cases, we were able to pinpoint the historical collection locality to within a radius of approximately 10 km, that is, for most towns and lakes. Larger geographic regions, for example, the Montreal metropolis, were assigned an imprecision of 100 km radius. Locality names that did not occur in our geographic gazeteer but that were nonetheless clearly Quebec locations were assigned a geoposition in the geographic centre of the province. These geographic coordinates were assigned a high level of imprecision (i.e. 1,000 km radius) and therefore should be filtered out of any data analysis that requires more specific locality data. Some place names refer to more than one locality and this is especially true for Quebec lakes (for example, the Canadian Geographic Names Data Base contains 144 Quebec entries for "*Lac Rond*"). We were sometimes able to establish which one was the correct collection locality (much research was conducted at the University of Montreal Laurentian Biological Research Station's *Lac Rond*), but otherwise we tried to be conservative by selecting a higher geographic level, most commonly the province itself. A certain number of geopositions can be refined in the future; these data will be updated and made available in the Canadensys.net datasets as time and resources allow. The geoposition coordinates were mapped with Simplmappr (Shorthouse 2010) to confirm that they all fell within the province of Quebec and to correct the two that did not.

## Geographic coverage

### Description

The specimen records are from the province of Quebec, Canada, comprising an area of approximately 1.5 million square km.

### Coordinates

44.99˚ and 62.59˚ Latitude; -57.10˚ and -79.76˚ Longitude.

## Taxonomic coverage

### Description

The specimen data records are all of the insect order Odonata, including 137 of the 150 species officially recorded from Quebec. The following list includes all 150 species. The total number of specimen records in the seven collections is in parentheses following each taxon name.

### Taxa included

**Table:** 

Rank	Scientific Name	
order	Odonata (40,447)	
suborder	Anisoptera (20,552)	Dragonflies
family	Aeshnidae Leach in Brewster, 1815 (4,339)	Darners
genus	*Aeshna* Fabricius, 1775 (3,690)	Mosaic darners
species	*Aeshna canadensis* Walker, 1908 (752)	Canada darner
species	*Aeshna clepsydra* Say, 1839 (6)	Mottled darner
species	*Aeshna constricta* Say, 1839 (119)	Lance-tipped darner
species	*Aeshna eremita* Scudder, 1866 (990)	Lake darner
species	*Aeshna interrupta* Walker, 1908 (583)	Variable darner
subspecies	*Aeshna interrupta interna* Walker, 1908 (82)	
subspecies	*Aeshna interrupta interrupta* Walker, 1908 (46)	
species	*Aeshna juncea* (Linnaeus, 1758) (60)	Sedge darner
species	*Aeshna septentrionalis* Burmeister, 1839 (3)	Azure darner
species	*Aeshna sitchensis* Hagen, 1861 (105)	Zigzag darner
species	*Aeshna subarctica* Walker, 1908 (157)	Subarctic darner
subspecies	*Aeshna subarctica subarctica* Walker, 1908 (16)	
species	*Aeshna tuberculifera* Walker, 1908 (83)	Black-tipped darner
species	*Aeshna umbrosa* Walker, 1908 (772)	Shadow darner
subspecies	*Aeshna umbrosa umbrosa* Walker, 1908 (29)	
species	*Aeshna verticalis* Hagen, 1861 (27)	Green-striped darner
genus	*Anax* Leach, 1815 (95)	Green darners
species	*Anax junius* (Drury, 1770) (95)	Common green darner
species	*Anax longipes* Hagen, 1861 (0)	Comet darner
genus	*Basiaeschna* Selys, 1883 (262)	Springtime darner
species	*Basiaeschna janata* (Say, 1839) (261)	Springtime darner
genus	*Boyeria* McLachlan, 1895 (151)	Spotted darners
species	*Boyeria grafiana* Williamson, 1907 (26)	Ocellated darner
species	*Boyeria vinosa* (Say, 1839) (121)	Fawn darner
genus	*Epiaeschna* Hagen, 1877 (23)	Swamp darner
species	*Epiaeschna heros* (Fabricius, 1798) (23)	Swamp darner
genus	*Gomphaeschna* Selys, 1871 (15)	Pygmy darners
species	*Gomphaeschna furcillata* (Say, 1839) (15)	Harlequin darner
genus	*Nasiaeschna* Selys in Förster, 1900 (0)	Cyrano darner
species	*Nasiaeschna pentacantha* (Rambur, 1842) (0)	Cyrano darner
genus	*Rhionaeshna* Förster, 1909 (0)	Neotropical darners
species	*Rhionaeshna mutata* (Hagen, 1861) (0)	Spatterdock darner
family	Cordulegastridae Hagen, 1877 (661)	Spiketails
genus	*Cordulegaster* Leach, 1815 (631)	Spiketails
species	*Cordulegaster diastatops* (Selys, 1854) (314)	Delta-spotted spiketail
species	*Cordulegaster maculata* Selys, 1854 (289)	Twin-spotted spiketail
species	*Cordulegaster obliqua* (Say, 1839) (22)	Arrowhead spiketail
family	Corduliidae Selys, 1850 (4,194)	Emeralds
genus	*Cordulia* Leach, 1815 (1,229)	Common emeralds
species	*Cordulia shurtleffi* Scudder, 1866 (1,211)	American emerald
genus	*Dorocordulia* Needham, 1901 (193)	Little emeralds
species	*Dorocordulia libera* (Selys, 1871) (193)	Racket-tailed emerald
genus	*Epitheca* Burmeister, 1839 (1,232)	Baskettails
species	*Epitheca canis* (McLachlan, 1886) (503)	Beaverpond baskettail
species	*Epitheca cynosura* (Say, 1839) (285)	Common baskettail
species	*Epitheca princeps* Hagen, 1861 (95)	Prince baskettail
species	*Epitheca spinigera* (Selys, 1871) (339)	Spiny baskettail
genus	*Helocordulia* Needham, 1901 (350)	Sundragons
species	*Helocordulia uhleri* (Selys, 1871) (349)	Uhler's sundragon
genus	*Neurocordulia* Selys, 1871 (20)	Shadowdragons
species	*Neurocordulia michaeli* Brunelle, 2000 (0)	Broad-tailed shadowdragon
species	*Neurocordulia yamaskanensis* (Provancher, 1875) (19)	Stygian shadowdragon
genus	*Somatochlora* Selys, 1871 (1,082)	Striped emeralds
species	*Somatochlora albicincta* (Burmeister, 1839) (232)	Ringed emerald
species	*Somatochlora brevicincta* Robert, 1954 (10)	Quebec emerald
species	*Somatochlora cingulata* (Selys, 1871) (191)	Lake emerald
species	*Somatochlora elongata* (Scudder, 1866) (184)	Ski-tipped emerald
species	*Somatochlora filosa* (Hagen, 1861) (1)	Fine-lined emerald
species	*Somatochlora forcipata* (Scudder, 1866) (29)	Forcipate emerald
species	*Somatochlora franklini* (Selys, 1878) (27)	Delicate emerald
species	*Somatochlora incurvata* Walker, 1918 (2)	Incurvate emerald
species	*Somatochlora kennedyi* Walker, 1918 (57)	Kennedy's emerald
species	*Somatochlora linearis* (Hagen, 1861) (0)	Mocha emerald
species	*Somatochlora minor* Calvert, 1898 (147)	Ocellated emerald
species	*Somatochlora septentrionalis* (Hagen, 1861) (10)	Muskeg emerald
species	*Somatochlora tenebrosa* (Say, 1839) (9)	Clamp-tipped emerald
species	*Somatochlora walshii* (Scudder, 1866) (65)	Brush-tipped emerald
species	*Somatochlora whitehousei* Walker, 1925 (1)	Whitehouse's emerald
species	*Somatochlora williamsoni* Walker, 1907 (104)	Williamson's emerald
genus	*Williamsonia* Davis, 1913 (3)	Boghaunters
species	*Williamsonia fletcheri* Williamson, 1923 (3)	Ebony boghaunter
family	Gomphidae Rambur, 1842 (2,266)	Clubtails
genus	*Arigomphus* Needham, 1897 (42)	Pond clubtails
species	*Arigomphus cornutus* (Tough, 1900) (21)	Horned clubtail
species	*Arigomphus furcifer* (Hagen, 1878) (21)	Lilypad clubtail
genus	*Dromogomphus* Selys, 1854 (112)	Spinylegs
species	*Dromogomphus spinosus* (Selys, 1854) (112)	Black-shouldered spinyleg
genus	*Gomphurus* Needham, 1901 (112)	Majestic clubtails
species	*Gomphurus fraternus* (Say, 1839) (24)	Midland clubtail
species	*Gomphurus vastus* (Walsh, 1862) (84)	Cobra clubtail
species	*Gomphurus ventricosus* (Walsh, 1863) (4)	Skillet clubtail
genus	*Hagenius* Selys, 1854 (49)	Dragonhunter
species	*Hagenius brevistylus* Selys, 1854 (49)	Dragonhunter
genus	*Hylogomphus* Needham, Westfall & May, 2000 (122)	Bantam clubtails
species	*Hylogomphus adelphus* (Selys, 1858) (122)	Mustached clubtail
genus	*Lanthus* Needham, 1897 (45)	Bantam clubtails
species	*Lanthus parvulus* (Selys, 1834) (44)	Northern pygmy clubtail
genus	*Ophiogomphus* Selys, 1854 (324)	Snaketails
species	*Ophiogomphus anomalus* Harvey, 1898 (6)	Extra-striped snaketail
species	*Ophiogomphus aspersus* Morse, 1895 (52)	Brook snaketail
species	*Ophiogomphus carolus* Needham, 1897 (40)	Riffle snaketail
species	*Ophiogomphus colubrinus* Selys, 1854 (147)	Boreal snaketail
species	*Ophiogomphus mainensis* Packard, 1863 (32)	Maine snaketail
species	*Ophiogomphus rupinsulensis* (Walsh, 1862) (36)	Rusty snaketail
genus	*Phanogomphus* Carle, 1986 (1,156)	American clubtails
species	*Phanogomphus borealis* (Needham, 1901) (95)	Beaverpond clubtail
species	*Phanogomphus descriptus* (Banks, 1896) (62)	Harpoon clubtail
species	*Phanogomphus exilis* (Selys, 1854) (572)	Lancet clubtail
species	*Phanogomphus lividus* (Selys, 1854) (2)	Ashy clubtail
species	*Phanogomphus spicatus* (Hagen in Selys, 1854) (425)	Dusky clubtail
genus	*Progomphus* Selys, 1854 (0)	Sanddragons
species	*Progomphus obscurus* (Rambur, 1842) (0)	Common sanddragon
genus	*Stylogomphus* Fraser, 1922 (58)	Least clubtails
species	*Stylogomphus albistylus* (Hagen in Selys, 1878) (58)	Eastern least clubtail
genus	*Stylurus* Needham, 1897 (185)	Hanging clubtails
species	*Stylurus amnicola* (Walsh, 1862) (10)	Riverine clubtail
species	*Stylurus notatus* (Rambur, 1842) (96)	Elusive clubtail
species	*Stylurus scudderi* (Selys, 1873) (55)	Zebra clubtail
species	*Stylurus spiniceps* (Walsh, 1862) (24)	Arrow clubtail
family	Libellulidae Leach in Brewster, 1815 (8,781)	Skimmers
genus	*Celithemis* Hagen, 1861 (14)	Small pennants
species	*Celithemis elisa* (Hagen, 1861) (11)	Calico pennant
species	*Celithemis eponina* (Drury, 1773) (3)	Halloween pennant
genus	*Erythemis* Hagen, 1861 (17)	Pondhawks
species	*Erythemis simplicicollis* (Say, 1839) (17)	Eastern pondhawk
genus	*Erythrodiplax* Brauer, 1868 (2)	Dragonlets
species	*Erythrodiplax berenice* (Drury, 1770) (2)	Seaside dragonlet
genus	*Ladona* Needham, 1899 (812)	Corporals
species	*Ladona julia* (Uhler, 1857) (812)	Chalk-fronted corporal
genus	*Leucorrhinia* Brittinger, 1850 (3,239)	Whitefaces
species	*Leucorrhinia frigida* Hagen, 1890 (83)	Frosted whiteface
species	*Leucorrhinia glacialis* Hagen, 1890 (1,147)	Crimson-ringed whiteface
species	*Leucorrhinia hudsonica* (Selys, 1850) (935)	Hudsonian whiteface
species	*Leucorrhinia intacta* (Hagen, 1861) (213)	Dot-tailed whiteface
species	*Leucorrhinia patricia* Walker, 1940 (26)	Canada whiteface
species	*Leucorrhinia proxima* Calvert, 1890 (822)	Belted whiteface
genus	*Libellula* Linnaeus, 1758 (613)	King skimmers
species	*Libellula incesta* Hagen, 1861 (16)	Slaty skimmer
species	*Libellula luctuosa* Burmeister, 1839 (51)	Widow skimmer
species	*Libellula pulchella* Drury, 1773 (127)	Twelve-spotted skimmer
species	*Libellula quadrimaculata* Linnaeus, 1758 (411)	Four-spotted skimmer
species	*Libellula semifasciata* Burmeister, 1839 (0)	Painted skimmer
genus	*Nannothemis* Brauer, 1868 (339)	Elfin skimmer
species	*Nannothemis bella* (Uhler, 1857) (339)	Elfin skimmer
genus	*Pachydiplax* Brauer, 1868 (6)	Blue dasher
species	*Pachydiplax longipennis* (Burmeister, 1839) (6)	Blue dasher
genus	*Pantala* Hagen, 1861 (31)	Rainpool gliders
species	*Pantala flavescens* (Fabricius, 1789) (26)	Wandering glider
species	*Pantala hymenaea* (Say, 1839) (5)	Spot-winged glider
genus	*Perithemis* Hagen, 1861 (0)	Amberwings
species	*Perithemis tenera* (Say, 1839) (0)	Eastern amberwing
genus	*Plathemis* Hagen, 1861 (258)	Whitetails
species	*Plathemis lydia* (Drury, 1770) (258)	Common whitetail
genus	*Sympetrum* Newman, 1833 (2,967)	Meadowhawks
species	*Sympetrum corruptum* (Hagen, 1861) (0)	Variegated meadowhawk
species	*Sympetrum costiferum* (Hagen, 1861) (298)	Saffron-winged meadowhawk
species	*Sympetrum danae* (Sulzer, 1776) (325)	Black meadowhawk
species	*Sympetrum internum* Montgomery, 1943 (297)	Cherry-faced meadowhawk
species	*Sympetrum obtrusum* Hagen, 1867 (1,428)	White-faced meadowhawk
species	*Sympetrum rubicundulum* (Say, 1839) (28)	Ruby meadowhawk
species	*Sympetrum semicinctum* (Say, 1839) (93)	Band-winged meadowhawk
species	*Sympetrum vicinum* (Hagen, 1861) (471)	Autumn meadowhawk
genus	*Tramea* Hagen, 1861 (1)	Saddlebags
species	*Tramea lacerata* Hagen, 1861 (1)	Black saddlebags
family	Macromiidae Needham, 1903 (311)	Cruisers
genus	*Didymops* Rambur, 1842 (230)	Brown cruisers
species	*Didymops transversa* (Say, 1839) (230)	Stream cruiser
genus	*Macromia* Rambur, 1842 (81)	River cruisers
species	*Macromia illinoiensis* Walsh, 1862 (80)	Swift river cruiser
suborder	Zygoptera (17,815)	Damselflies
family	Calopterygidae Selys, 1850 (1,588)	Broad-winged damsels
genus	*Calopteryx* Leach, 1815 (1,483)	Jewelwings
species	*Calopteryx aequabilis* Say, 1839 (363)	River jewelwing
species	*Calopteryx amata* Hagen, 1889 (308)	Superb jewelwing
species	*Calopteryx maculata* (Beauvois, 1805) (750)	Ebony jewelwing
genus	*Hetaerina* Hagen in Selys, 1853 (31)	Rubyspots
species	*Hetaerina americana* (Fabricius, 1798) (10)	American rubyspot
family	Coenagrionidae Kirby, 1890 (11,583)	Pond damsels
genus	*Amphiagrion* Selys, 1876 (97)	Red damsels
species	*Amphiagrion saucium* (Burmeister, 1839) (97)	Eastern red damsel
genus	*Argia* Rambur, 1842 (365)	Dancers
species	*Argia apicalis* (Say, 1839) (0)	Blue-fronted dancer
species	*Argia fumipennis* (Burmeister, 1839) (109)	Variable dancer
subspecies	*Argia fumipennis violacea* (Hagen, 1861) (109)	
species	*Argia moesta* (Hagen, 1861) (250)	Powdered dancer
genus	*Chromagrion* Needham, 1903 (404)	Aurora damsel
species	*Chromagrion conditum* (Hagen in Selys, 1876) (404)	Aurora damsel
genus	*Coenagrion* Kirby, 1890 (1,010)	Eurasian bluets
species	*Coenagrion interrogatum* (Hagen in Selys, 1876) (591)	Subarctic bluet
species	*Coenagrion resolutum* (Hagen in Selys, 1876) (419)	Taiga bluet
genus	*Enallagma* Charpentier, 1840 (6,873)	American bluets
species	*Enallagma anna* Williamson, 1900 (0)	River bluet
species	*Enallagma annexum* (Hagen, 1861) (632)	Northern bluet
species	*Enallagma antennatum* (Say, 1839) (131)	Rainbow bluet
species	*Enallagma aspersum* (Hagen, 1861) (387)	Azure bluet
species	*Enallagma boreale* (Selys, 1875) (2,744)	Boreal bluet
species	*Enallagma carunculatum* Morse, 1895 (190)	Tule bluet
species	*Enallagma civile* (Hagen, 1861) (29)	Familiar bluet
species	*Enallagma clausum* Morse, 1895 (18)	Alkali bluet
species	*Enallagma ebrium* (Hagen, 1861) (747)	Marsh bluet
species	*Enallagma exsulans* (Hagen, 1861) (142)	Stream bluet
species	*Enallagma geminatum* Kellicott, 1895 (30)	Skimming bluet
species	*Enallagma hageni* (Walsh, 1863) (1,361)	Hagen's bluet
species	*Enallagma signatum* (Hagen, 1861) (101)	Orange bluet
species	*Enallagma traviatum* (Selys, 1876) (0)	Slender bluet
species	*Enallagma vernale* Gloyd, 1943 (221)	Vernal bluet
species	*Enallagma vesperum* Calvert, 1919 (89)	Vesper bluet
genus	*Ischnura* Charpentier, 1840 (1,289)	Forktails
species	*Ischnura hastata* (Say, 1839) (0)	Citrine forktail
species	*Ischnura posita* (Hagen, 1861) (8)	Fragile forktail
species	*Ischnura verticalis* (Say, 1839) (1,277)	Eastern forktail
genus	*Nehalennia* Selys, 1850 (1,455)	Sprites
species	*Nehalennia gracilis* Morse, 1895 (583)	Sphagnum sprite
species	*Nehalennia irene* (Hagen, 1861) (866)	Sedge sprite
family	Lestidae Calvert, 1901 (4,644)	Spreadwings
genus	*Lestes* Leach, 1815 (4,472)	Pond spreadwings
species	*Lestes congener* Hagen, 1861 (695)	Spotted spreadwing
species	*Lestes disjunctus* Selys, 1862 (2,187)	Northern spreadwing
species	*Lestes dryas* Kirby, 1890 (376)	Emerald spreadwing
species	*Lestes eurinus* Say, 1839 (470)	Amber-winged spreadwing
species	*Lestes forcipatus* Rambur, 1842 (256)	Sweetflag spreadwing
species	*Lestes inaequalis* Walsh, 1862 (22)	Elegant spreadwing
species	*Lestes rectangularis* Say, 1839 (133)	Slender spreadwing
species	*Lestes unguiculatus* Hagen, 1861 (298)	Lyre-tipped spreadwing
species	*Lestes vigilax* Hagen in Selys, 1862 (15)	Swamp spreadwing

## Temporal coverage

**Data range:** 1875-6-08 – 2015-6-24.

## Usage rights

### Use license

Creative Commons Public Domain Waiver (CC-Zero)

## Data resources

### Data package title

Quebec Odonata specimen data

### Number of data sets

1

### Data set 1.

#### Data set name

Quebec Odonata specimen data

#### Data format

Darwin Core

#### Number of columns

49

#### Character set

UTF-8

#### Description

The dataset contains the specimen-level metadata for Quebec Odonata as captured from seven publicly-accessible entomological collections in Quebec (Suppl. material 1). Future updates will be available from each collection at Canadensys.net.

**Data set 1.** 

Column label	Column description
occurrenceID	The globally unique identifier for the record.
type	The nature or genre of the resource, i.e. "PhysicalObject".
modified	The most recent date on which the resource was changed.
language	The language of the resource, i.e. English and/or French, "en|fr".
licence	The legal document giving official permission to do something with the resource. i.e. "http://creativecommons.org/publicdomain/zero/1.0/legalcode".
rightsHolder	The organisation owning or managing rights over the resource, e.g. "Université de Montréal".
bibliographicCitation	A bibliographic reference for the resource as a statement indicating how this record should be cited (attributed) when used, e.g. "QMOR1"
collectionID	An LSID for the collection or dataset from which the record was derived, e.g. "urn:lsid:biocol.org:col:34164".
datasetID	The DOI for the original Canadensys source of the data, e.g. "10.5886/qwvt63fz".
institutionCode	The name of the institution having custody of the object(s) or information referred to in the record, e.g. "Université de Montréal".
collectionCode	The coden identifying the collection or dataset from which the record was derived, e.g. "QMOR".
datasetName	The name identifying the dataset from which the record was derived, e.g. "Ouellet-Robert Entomological Collection".
basisOfRecord	The specific nature of the data record, i.e. "PreservedSpecimen".
catalogNumber	An identifier for the record within the dataset or collection, e.g. "QMOR1.001", where "QMOR1" refers to the museum object (e.g. the vial or envelope) and ".001" refers to one or several specimens contained in that museum object.
recordedBy	The primary collector or collectors of the specimen(s), e.g. "Robert, Adrien".
individualCount	The number of individuals represented in the data record.
sex	The sex of the biological individual(s) represented by the specimens, i.e. "Male" or "Female".
lifeStage	The age class or life stage of the biological individual(s), i.e. "Adult", "Immature", "Exuvium" or "Egg".
preparations	The preparation and preservation method for the specimens, i.e. "Envelope", "Pin", "Vial" or "Pill box".
otherCatalogNumbers	An identifier for the museum object within the dataset or collection, e.g. "QMOR1". See catalogNumber.
eventDate	The date or interval during which the collection event occurred, e.g. "2012-01-05".
startDayOfYear	The first possible day of the year that the collection event occurred, i.e. between 1 and 365.
endDayOfYear	The last possible day of the year that the collection event occurred, i.e. between 1 and 365.
year	The four-digit year in which the collection event occurred, according to the Common Era Calendar, i.e. between 1875 and 2015.
month	The ordinal month in which the collection event occurred, i.e. between 1 and 12.
day	The integer day of the month on which the collection event occurred, i.e. between 1 and 31.
continent	The name of the continent on which the collection occurred, i.e. "North America".
country	The name of the country in which the collection occurred, i.e. "Canada".
stateProvince	The name of the next smaller administrative region than country (state, province, canton, department, region etc.) in which the collection occurred, i.e. "Quebec".
locality	The specific description of the place. This term may contain information modified from the original to correct perceived errors or standardise the description, e.g. "Saint-Hippolyte, Station de Biologie des Laurentides de l'Université de Montréal, Route de la station".
decimalLatitude	The geographic latitude in decimal degrees of the geographic centre of a Location. Positive values are north of the Equator, negative values are south of it.
decimalLongitude	The geographic longitude in decimal degrees of the geographic centre of a Location. Positive values are east of the Greenwich Meridian, negative values are west of it.
coordinateUncertaintyInMetres	The horizontal distance in metres from the given decimalLatitude and decimalLongitude describing the smallest circle containing the whole of the Location.
georeferenceSources	A list (concatenated and separated) of maps, gazetteers or other resources used to georeference the Location, i.e. "Canadian Geographic Names Data Base", "Google Maps", "Google Earth".
identifiedBy	The primary determiner or determiners of the specimen(s), e.g. "Robert, Adrien".
dateIdentified	The date (year) on which the specimen was determined, e.g. "1972".
scientificName	The full scientific name, as given by the determiner, with authorship and date information if known, e.g. "Gomphus descriptus Banks, 1896".
acceptedNameUsage	The full name, with authorship and date information if known, of the currently valid name of the taxon, e.g. "Phanogomphus descriptus (Banks, 1896)".
kingdom	The full scientific name of the kingdom in which the taxon is classified, i.e. "Metazoa".
phylum	The full scientific name of the phylum or division in which the taxon is classified, i.e. "Arthropoda".
class	The full scientific name of the class in which the taxon is classified, i.e. "Insecta".
order	The full scientific name of the order in which the taxon is classified, i.e. "Odonata".
family	The full scientific name of the family in which the taxon is classified, e.g. "Aeshnidae".
genus	The full scientific name of the genus in which the taxon is classified, e.g. "Aeshna".
specificEpithet	The name of the species epithet of the scientificName, e.g. "interrupta".
infraspecificEpithet	The name of the subspecific epithet of the scientificName, e.g. "interna".
taxonRank	The taxonomic rank of the most specific name in the scientificName, i.e. "Family", "Genus", "Species" or "Subspecies".
scientificNameAuthorship	The authorship information for the scientificName formatted according to the conventions of the applicable nomenclaturalCode, e.g. "Walker, 1908".
nomenclaturalCode	The code of nomenclature that governs the scientificName, i.e. "ICZN", the International Code of Zoological Nomenclature.

## Additional information

**Figure 4.**
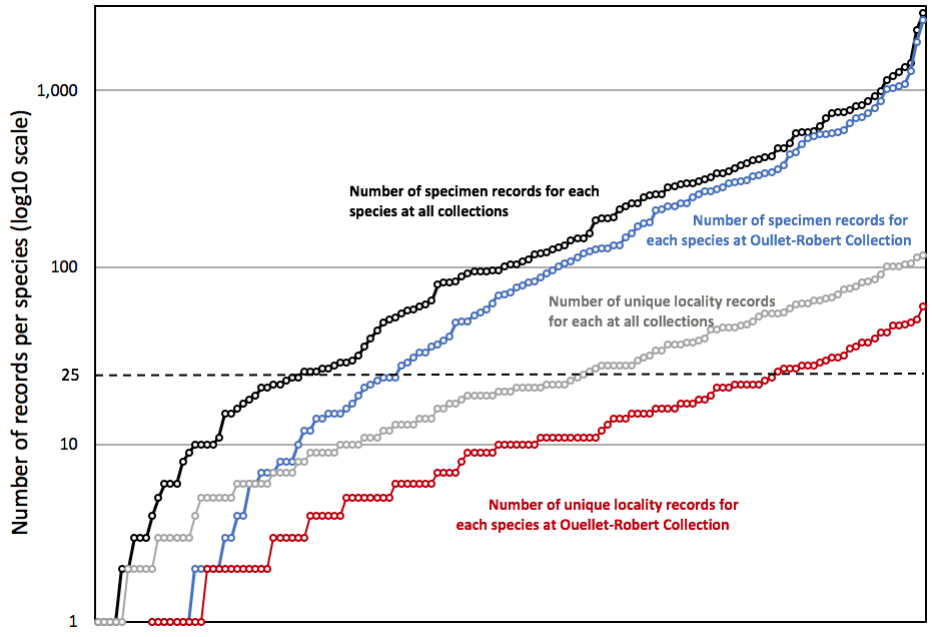
Number of specimen occurrence and unique locality records per species for 137 Quebec Odonata species found in all seven collections (black and grey lines) and 128 species found in the Ouellet-Robert Collection (blue and red lines). The dashed line indicates the conservative 25-record threshold calculated by van Proosdij et al. (2015) for developing species distribution models.

**Figure 5.**
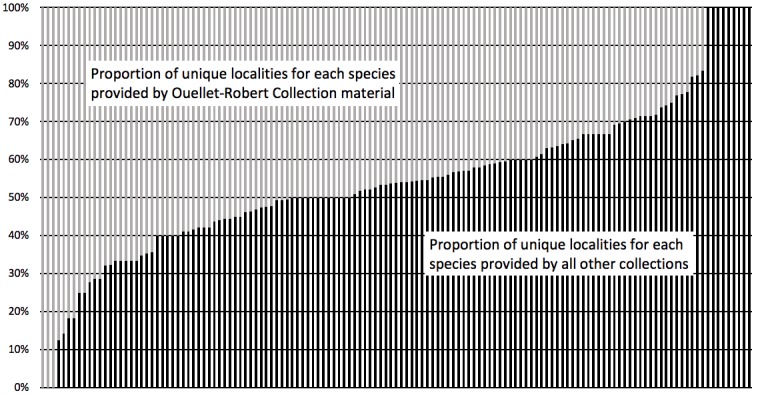
Proportion of unique localities per species provided by Ouellet-Robert Collection specimens versus material provided by all other collections. The first three species are those present at the Ouellet-Robert Collection, but absent elsewhere and the last nine are those present elsewhere, but absent from the Ouellet-Robert Collection.

**Figure 6.**
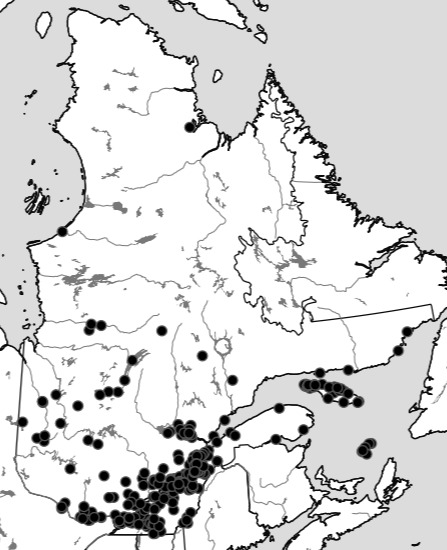
Collection localities of the Laval University specimens.

**Figure 7.**
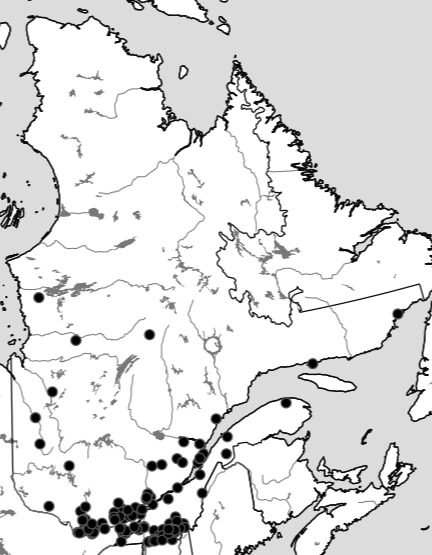
Collection localities of the Insectarium of Montreal specimens.

**Figure 8.**
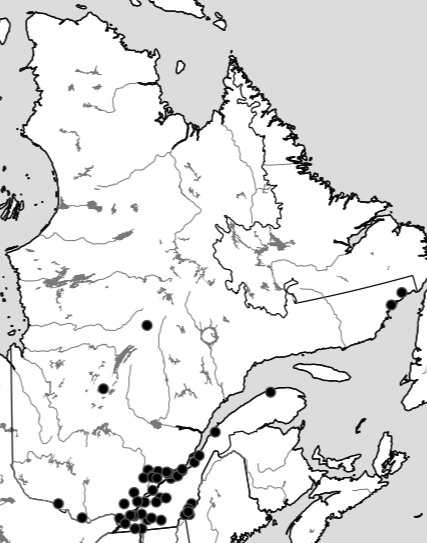
Collection localities of the Quebec Insect Collection specimens.

**Figure 9.**
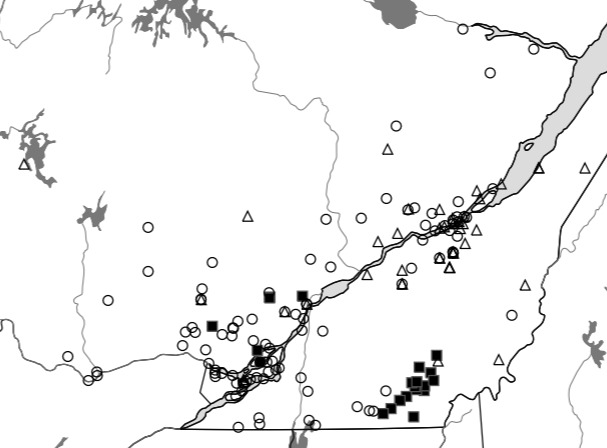
Collection localities of the Lyman Museum (circles), Bishop's University (squares) and Insectarium René-Martineau (triangles) specimens, concentrated in the regions of Montreal, Sherbrooke and Quebec City, respectively.

Although the Ouellet-Robert Collection accounted for 81% of all the species-level occurrence records (Fig. 4, Table 1), these specimens were collected in a relatively small number of localities, averaging only 45% of all unique localities per species (Figs 1, 5). Adding the other six collections dramatically increased the geographic coverage, especially the University of Laval Collection with as many localities as the Ouellet-Robert Collection (Table 1), including a nice series of specimens from Anticosti Island in the Gulf of Saint Lawrence (Fig. 6). The Insectarium of Montreal and Quebec Insect Collection both have broad geographic sampling (Figs 7, 8), whereas that of the other three collections is narrower, concentrated near Montreal, Sherbrooke and Quebec City (Fig. 9).

**Figure 10.**
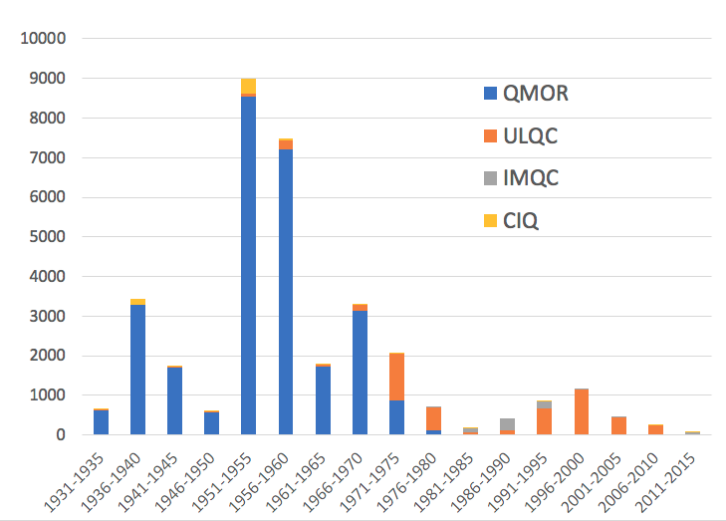
Number of species-level occurrence records from the four largest collections collected in each five-year interval.

Adding the other collections also increased the taxonomic coverage. Whereas specimens of three species are held only at the Ouellet-Robert Collection, nine species, absent from this collection, are held elsewhere (Fig. 5), including four held only in a single other collection (Table 1). In all, seven species are present in a single collection each, whereas only seven are present in all seven collections (Table 2). The temporal coverage also broadened considerably with the addition of the other six collections. Whereas overall sampling is dominated by the Ouellet-Robert Collection, mostly thanks to the efforts of its long-time curator, Adrien Robert (Robert 1963), there is almost no material from the mid-1970s onwards (Fig. 10). It is the Laval University Collection that provides the vast majority of the material collected during the 1970s and then especially from the 1990s to 2010. This latter collection is in especially nice curatorial condition thanks to the work of Jean-Marie Perron.

The volume of material in the combined dataset should be useful for future modelling and other distribution-related analyses. More than half of the species are represented by over 100 specimens in the combined dataset (Fig. 4). If we restrict future work to only those species for which we have the conservative estimate of 25 unique records suggested by van Proosdij et al. (2015), 25 species at the Ouellet-Robert Collection and 57 across all collections are suitable for distribution modelling (Fig. 4). Using this admittedly somewhat arbitrary metric, the additional 19% of material gained by digitising the Odonata in the six other collections represents a two-fold improvement.

## Supplementary Material

64DC0014-D9FF-541A-91BD-1EAB7AA6D4C8 10.3897/BDJ.8.e49450.suppl1 Supplementary material 1 Quebec Odonata specimen data Data type: occurences
Brief description: Specimen metadata as of 16-12-2019, in Darwin Core format, for the Quebec Odonata specimens deposited in seven publicly-accessible research collections in Quebec, Canada.
File: oo_365494.csv https://binary.pensoft.net/file/365494 Favret C, Boucher S, Cloutier C, Cloutier L, Emond MC, Harper PP, Moisan-De Serres J, Larrivée M, Pelletier G, Perron JM, Piché C, Savage J, Wagner G, Wheeler T

## Figures and Tables

**Table 1.** Summary of Quebec insect research collections and their Quebec Odonata holdings.

Institution	Collection	Location	Canadensys DOI	No. occurrence records	No. species-level records	No. species	No. unique species	No. species-level collection localities
University of Montreal	Ouellet-Robert Collection (QMOR)	Montreal	10.5886/qwvt63fz	31,595	29,982	128	3	222
Laval University	Collection entomologique de l'Université Laval (ULQC)	Quebec City	10.5886/bxbpry	4,994	4,993	122	2	296
McGill University	Lyman Entomological Museum (LEMQ)	Sainte-Anne-de-Bellevue	10.5886/q79vhp1e	1,841	270	45		39
Insectarium of Montreal	(IMQC)	Montreal	10.5886/i6z1vo	922	782	110	1	109
Government of Quebec	Collection d'Insectes du Québec (CIQ)	Quebec City	10.5886/msuujw	655	653	71	1	59
Bishop's University	Bishop's University Insect Collection (BUIC)	Sherbrooke	10.5886/nmcxfj	228	62	19		14
Laurentian Forestry Centre	Insectarium René-Martineau (IRM)	Quebec City	10.5886/d6vnc2	212	209	50		43
		**TOTAL**		40,459	36,963	137	7	616

**Table 2.** Number of Quebec Odonata species deposited in no collection (absent from all collections), a single collection, two to six collections or all seven collections.

Number of collections	Number of species
No collections	13
1 collection	7
2 collections	21
3 collections	30
4 collections	28
5 collections	19
6 collections	25
All 7 collections	7
TOTAL	150
